# Right Heart Catheterization—Background, Physiological Basics, and Clinical Implications

**DOI:** 10.3390/jcm8091331

**Published:** 2019-08-28

**Authors:** Grzegorz M. Kubiak, Agnieszka Ciarka, Monika Biniecka, Piotr Ceranowicz

**Affiliations:** 1 Department of Cardiac, Vascular and Endovascular Surgery and Transplantology, Silesian Centre for Heart Diseases, Medical University of Silesia, 41-800 Zabrze, Poland; 2 Department of Cardiovascular Diseases, University Hospitals Leuven, University of Leuven, Herestraat 49, Leuven 3000, Belgium; 3 KardioMed Silesia, M. Curie-Skłodowskiej 10C, 41-800 Zabrze, Poland; 4 Department of Physiology, Faculty of Medicine, Jagiellonian University Medical College, 31-008 Kraków, Poland

**Keywords:** right heart catheterization, pulmonary hypertension, heart failure, diagnosis, prognostic evaluation, clinical implications

## Abstract

The idea of right heart catheterization (RHC) grew in the milieu of modern thinking about the cardiovascular system, influenced by the experiments of William Harvey, which were inspired by the treatises of Greek philosophers like Aristotle and Gallen, who made significant contributions to the subject. RHC was first discovered in the eighteenth century by William Hale and was subsequently systematically improved by outstanding experiments in the field of physiology, led by Cournand and Dickinson Richards, which finally resulted in the implementation of pulmonary artery catheters (PAC) into clinical practice by Jeremy Swan and William Ganz in the early 1970s. Despite its premature euphoric reception, some further analysis seemed not to share the early enthusiasm as far as the safety and effectiveness issues were concerned. Nonetheless, RHC kept its significant role in the diagnosis, prognostic evaluation, and decision-making of pulmonary hypertension and heart failure patients. Its role in the treatment of end-stage heart failure seems not to be fully understood, although it is promising. PAC-guided optimization of the treatment of patients with ventricular assist devices and its beneficial introduction into clinical practice remains a challenge for the near future.

## 1. Introduction

### 1.1. Aim of the Review

To elucidate the state of the art of right heart catheterization, focusing on the background of the initial experiments and its physiological meaning, which leads to its implementation in clinical practice. To indicate potential directions for future research.

### 1.2. From Greek Philosophy into Modern Understanding

Right heart catheterization (RHC) has emerged as an invasive procedure with a potent role among the current armory of clinical diagnostic tools, including computed tomography (CT), magnetic resonance imaging (MRI), and genetical immunoassays sampling [1,2]. The birth of this technique is strictly associated with the genial English physiologist and physician William Harvey (1578–1657), who actually proposed a theoretical model of the functioning of an animal circulatory system, which he unveiled in the work entitled Exercitatio Anatomica de Motu Cordis et Sanguinis in Animalibus and published in 1628 in Frankfurt [3].

The work, containing 17 chapters within 72 pages, irretrievably changed the modern understanding of the blood circulation. It is important to keep in mind that Galen was the first person to make extraordinary contributions to the concepts of physiology and medicine that turned out to be widely accepted paradigms. He believed that the circulatory system consisted of two separated parts: venous, originating from the liver, and arterial, originating from the heart. The idea of two separate, unidirectional systems of distribution rather than a unified system of circulation remained unchallenged for many centuries. Harvey, although heavily influenced by his potent predecessor, unwillingly found himself in the position of a revolutionary antagonizer. Harvey’s way of thinking and analyzing different phenomena was heavily influenced by another ancient Greek philosopher, Aristotle [4,5]. The Aristotelian way of thinking would be to engage not only rigorous mathematical calculations but also analogies to the natural phenomena covered by physics, biology, and other empirical sciences.

“De motu cordis” was released in Europe during the Thirty Years’ War, described as one of the most destructive and violent periods in human history. This era was characterized by different political, religious, and cultural movements (the Reformation, Counter-reformation, and forming of the Anglican Church). During these times of overwhelming crime, plagues, and the rising wave of inquisition, the signs of independent thinking, especially when based on evidence and experiments, were unwanted and considered dangerous [6,7].

### 1.3. Pioneers of Right Heart Catheterization

As time passed, further attempts to experimentally evaluate the circulatory system were made. In 1711, Stephen Hale reported the measurement of cardiac output (CO) with the use of brass pipes introduced into equine veins and arteries [8,9,10,11]. The technical aspects of these examinations were further improved by Claude Bernard, who challenged the pulmonary combustion model of circulation, claiming that the blood temperature varies significantly between the systemic and pulmonary circulatory system [12]. Since his previous attempts to encounter surgical techniques in the temperature measurement appeared to be inaccurate, Bernard developed the technique of exposing the jugular vein and carotid artery of a horse in order to perform the measurements above. Bernard proved the technical feasibility of those examinations, which were continued by another two French physiologists, Baptiste Chauveau and Étienne Marey [13,14,15]. The collaboration of these has been linked to the virtual beginning of intracardiac pressure recordings.

The innovative dual-lumen catheter device was designed to simultaneously assess the intracardiac pressures of the left ventricle and the aorta, as well as the atrium and the right ventricle. These experiments discovered the so far unreported phenomena of ventricular pressure curves, isometric phase of ventricular contraction, interventricular synchrony, and the chronology of valve closing. It is worth noting that Marey was also renowned as a pioneer of electrocardiography, with significant contributions in the field made by the discovery of the refractory period in 1875 and the first recording of electrocardiogram with the use of capillary electrometer in 1876 [16].

Remarkably, it was nearly three decades before Willem Einthoven reported his invention, which was about to revolutionize the modern diagnosis and treatment of cardiovascular disorders. In his late years, Marey, being somehow possessed by the idea of a general visualization of the motion of different species present in the environment, turned his professional activity towards chronophotography, which he documented in books entitled *Le mouvement* and *Le vol des oiseaux* [17].

### 1.4. Nobel Prize for the Famous Three

German scientists Fritz Bleichroeder, Ernst Unger, and W. Loeb continued the pathway of right heart catheterization in animals, and performed self-experiments in that field; however, these were scarcely documented [18]. A breakthrough came when a recently graduated doctor Werner Forssmann performed the first in-vivo catheterization of his own heart, which took place in Eberswalde in 1929 [19,20,21,22,23,24]. This experiment was an act of bravery which took the human cardiac catheterization from theory into practice in a short period of time. Forssmann’s attitude towards the National Socialist German Workers’ Party (NSDAP) arose from the phase of an early euphoria into the late deception and was basically indifferent from the typical attitude of his fellow countrymen [25].

The denazification process, to which he had been submitted in the late 1940s, did not alter his recognition in the area of right catheterization, for which he was awarded the Nobel Prize in 1956, along with Doctors André Cournand and Dickinson Richards. Remarkably, Doctors Cournand and Richards, during their work at the Department of Physiology of the Bellevue Hospital in New York, made an extraordinary contribution to the refinement of the antecedents techniques; they were the first to report a reliable measurement of CO using the Fick principle [26].

### 1.5. Jeremy Swan and William Ganz—Catheterization in Clinical Practice

Moreover, they understood the phenomenon of oxygen debt in the setting of “clinical shock”, which they managed to treat successfully with the implementation of a human serum albumin [27]. These observations set the stage for future scientific research in the field of different kinds of shock, their risk stratification and management [28,29]. Interestingly, Cournand positively verified the usefulness of a ballistocardiogram and its correlation with the results of invasive measurement of CO performed using the Fick principle [30]. Having proven that the catheter might be uneventfully left in the right heart cavities for a prolonged period, exceeding twenty-four hours, some further improvements were made, which eventually evolved into the discovery of the pulmonary capillary/artery wedge pressure (PCWP/PAWP).

**Figure 1:**
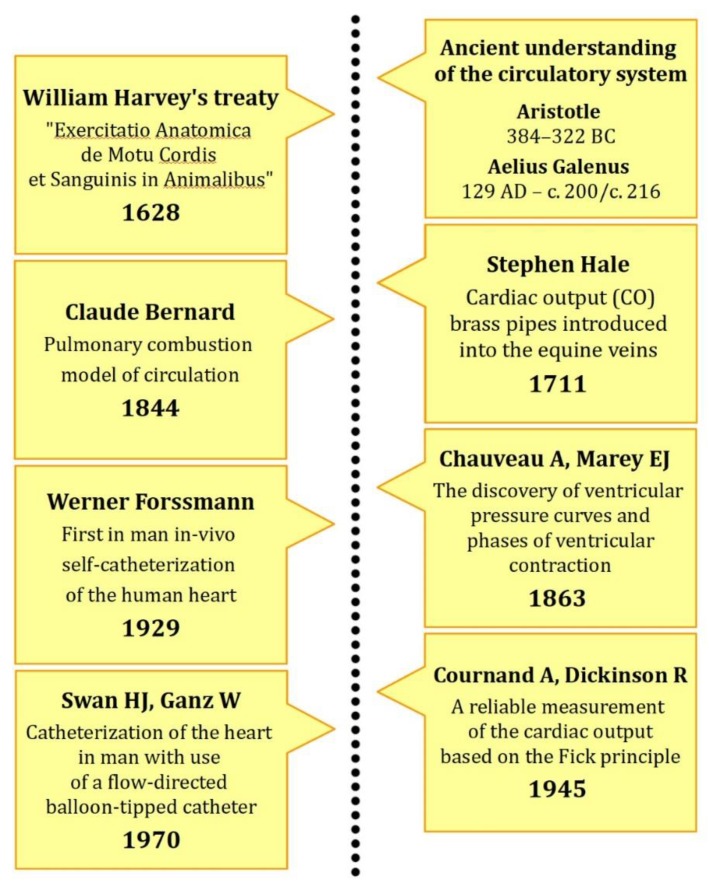
The milestones of right heart catheterization (RHC).

Its monitoring, first demonstrated by Hellems and co-workers, was a fairly safe technique of estimating left atrial pressure in the absence of mitral regurgitation [31]. Remarkably, another innovation was associated with the introduction of a balloon at the end of the tip, whose presence facilitated migration with the blood flow [32,33]. Another improvement was associated with the utilization of the thermodilution method to precisely assess CO. Needless to say, it added important information that, along with the modifications above, appears safe and reliable [34,35]. To see the milestones of RHC, please refer to Figure 1.

### 1.6. Technique of the Measurement and Basic Definitions

The preferred access point is the internal jugular vein. Its patency and anatomical conditions are reassured during an ultrasound examination, which is believed to reduce access site complications [36,37,38,39]. The currently used Seldinger technique is based on the principle of a guiding wire that enters into the vessel lumen. After an anatomical evaluation of a correct position, a special, dedicated sheath with a valve is inserted into the lumen, enabling easy access into the internal jugular vein and the subsequent structures [40,41,42,43]. A Swan-Ganz (S-G) catheter, basically not different from the prototype used by its inventors in 1970, is inserted via the internal jugular vein into the right atrium, ventricle, and pulmonary arteries. In some cases, RHC might be technically demanding, particularly in individuals with severely dilated right ventricle (RV) due to pulmonary hypertension, severe tricuspid regurgitation (TR), or other obstacles located in the right heart, like pacemaker leads or artificial valves. In these cases, the use of fluoroscopic guidance or even the angioplasty wire in order to acquire an adequate PAWP may be beneficial. An unreliable PAWP value may be verified by a direct left ventricular end-diastolic pressure (LVEDP) measurement via the arterial access [44]. Several potential pitfalls may occur during RHC. It should be underlined that PAWP is vulnerable to errors in parameter of key importance in the diagnosis of pulmonary hypertension (PH). It might be either over- or, more often, under-wedged, which may lead to a misdiagnosis and, consequently, inappropriate treatment [45]. Since the PAWP is heavily dependent on respiration, it is a subject of debate whether it should be measured at the end-expiration or averaged during spontaneous breathing [44,45]. It applies mainly to the patients with respiratory disorders, in whom there is an intrathoracic pressure swing influencing the intracardiac conditions [45].

## 2. Physiological Aspects of Right Heart Catheterization

**Figure 2:**
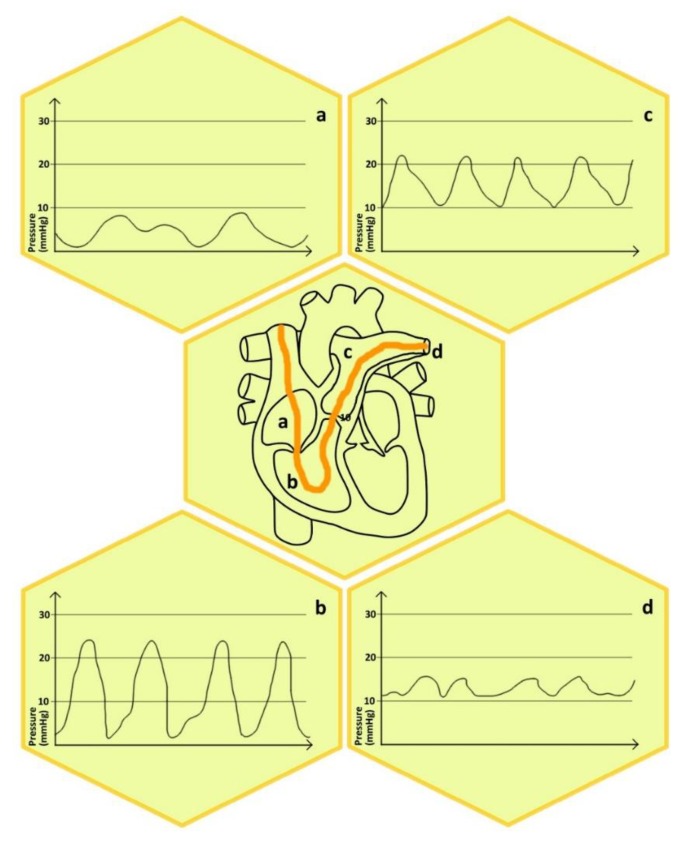
Typical pressure curves and values assessed during RHC within the physiological states. (**a**) right atrium, (**b**) right ventricle, (**c**) pulmonary artery, (**d**) tip of the catheter in a small artery.

The European Society of Cardiology (ESC) and the European Respiratory Society (ERS) recommend performing the zeroing of the transducer at the mid-thoracic line (with the reference point defined by the crossing of the frontal plane at the mid-thoracic level, the transverse plane at the level of the fourth anterior intercostal space, and the midsagittal plane in a supine patient halfway between the anterior sternum and the bed surface [1,46]). It is important to emphasize the role of an adequate calibration of the pressure transducer; however, the theoretical and experimental background of right ventricle elasticity, along with the associations concerning volumes in different clinical states and different species, falls beyond the scope of the current review [47,48,49,50,51,52,53,54,55,56]. For research purposes, it is recommended to perform a single-beat estimation of the right ventricular end-systolic pressure–volume relationship [57,58,59]. To see the typical pressure curves and values assessed during RHC within the physiological states please refer to Figure 2.

After zeroing, the measurements of the right atrium, right ventricle, pulmonary artery, and capillary wedge pressure are performed. The currently accepted and recommended measurement of cardiac output is the thermodilution method, in which a constant amount of saline (usually 10 cL) is injected into the bloodstream—the difference in the temperature is analyzed by the thermistor and recorded. It is important to collect at least three to five results of CO in order to acquire reliable values, especially given that it affects the decision-making process in the real-life clinical setting (eligibility for heart transplantation, mechanical circulatory support–mono/bi–ventricular assist device). It is noteworthy that inaccurately measured, reduced CO will result in raised pulmonary vascular resistance (PVR), according to the equation (PAPm (PAPm—pulmonary artery pressure mean)—PAWP/CO = PVR). In clinical practice, the assessment of the CO by the use of thermodilution is preferred to the evaluation by the Fick method. The latter, in which the cardiac output is calculated as the quotient of oxygen uptake (O2) and the difference of the arterial and mixed venous oxygen content, is advised in the case of tricuspid regurgitation. The main limitation of the Fick method is caused by the fact that the bedside measurement of oxygen uptake is technically demanding. To see the basic definitions and range of normal values please refer to Table 1.

## 3. Clinical Practice Implications

### 3.1. Safety of Right Heart Catheterization—The Evaluation of Clinical Outcomes

Since there existed a vital need to monitor the severely ill patients’ pulmonary artery, catheterization has been widely introduced into clinical practice, including pediatrics, cardiac surgery, invasive treatments in different kinds of clinical settings, including a wide variety of shock states originating from the circulatory and/or septic background [60,61,62,63,64,65,66,67,68]. The use of this technology, mainly without appropriate guidance or experience, resulted in few complications, reported thereafter in the form of case reports [69,70,71,72,73,74,75].

These complications had different natures—from relatively mild dissections in the puncture site to serious ones with systemic clinical implications, like tricuspid valve chord rupture or pulmonary hemorrhages. Apparently, the learning curve and equipment imperfections could have been blamed to some extent; however, re-evaluation of this clinical modality of invasive cardiovascular system assessment, according to the evidence-based medicine, rapidly generated interest in the community of intensive care practitioners and physicians [76,77,78,79,80,81,82].

The Study to Understand Prognoses and Preferences for Outcomes and Risks of Treatments (SUPPORT) lead by Connors and co-investigators was the first prospective cohort study designed to address the issue of pulmonary artery catheter (PAC) effectiveness and costs [76]. PAC (formerly called the Swan-Ganz catheter) did not have any favorable effect, moreover it significantly raised the cost of treatment. This work has been succeeded by others, like Wheeler et al., who evaluated one thousand patients with acute lung injuries and stated that PAC did not have a positive influence on the course of the management process in comparison to the central venous catheter and should, therefore, not be used in this clinical condition [77]. Harvey and co-workers did not report any benefit from the PAC-guided treatment in severely ill patients enrolled within the “PAC-man” trial in the United Kingdom [78]. Ramsey et al. [82] retrospectively analyzed the outcomes of PAC use in non-emergent coronary artery bypass graft surgery and stated that not only did PAC not have any beneficial effect in reducing complications, but it was also associated with a two-fold increase in mortality, extended the length of hospital stay, and significantly raised the costs [82]. It referred predominantly to the low volume centers in which the PAC-derived data were apparently inadequately interpreted. These studies gradually changed the clinical practice in the USA, resulting in a decrease in PAC use in response, which was reported by Wiener and Ikuta [83,84].

### 3.2. The Position of Right Heart Catheterization in the Diagnostic and Management Algorithm of Heart Failure

RHC is currently often used to monitor patients and guide the therapy to optimize filling pressures and volume overloads. Some centers use it excessively in almost all patients, highlighting the point that this technique is basically less prone to subjective inter-observer variability. The Evaluation Study of Congestive Heart Failure and Pulmonary Artery Catheterization Effectiveness (ESCAPE) (85) investigators assessed the results of using RHC in 433 patients enrolled randomly in either an interventional or conservative arm. Remarkably, RHC was used intentionally to optimize pharmacological treatment, not only for those that did not prove any benefit; additionally, it was associated with a higher risk of adverse events, predominantly PAC infection [85]. Sionis [86] and co-workers analyzed the data of cardiogenic shock (CS) patients included in the CardShock registry. Eighty-two of them (37.4%) diagnosed with PAC received goal-oriented therapy consisting of mechanical ventilation, renal replacement therapy, and mechanical assist devices more often as compared to the routinely assessed group of patients (*p* < 0.01). Nevertheless, PAC use was not associated with any survival benefit within a 30-day observation period, even after propensity score-matching analysis. Doshi et al. [87] compared the trends in the utilization of PAC between 2005 and 2014 in a total of over six million of patients with heart failure (HF) with reduced or preserved ejection fraction (HFrEF/HFpEF respectively). They found that the PAC utilization per 1000 hospitalizations significantly declined from 2005 to 2010 in both HFrEF and HFpEF. On the contrary, from 2010 to 2014, the use of PAC per 1000 hospitalizations increased in both HFrEF and HFpEF. The temporary decline in risk-adjusted mortality during the study period for HFrEF and HFpEF is mostly associated with the improvements in HF therapies. Hernandez [88] and co-authors retrospectively analyzed the trends in PAC utilization between 2004 and 2014 in a total of 9,431,944 patients admitted with the primary diagnosis of HF (*n* = 8,516,528), or who developed CS (*n* = 915,416) during the index hospitalization. They observed a decline in the use of PAC in patients with HF, from 8 PAC used per 1000 admissions in 2004 to 6 PAC used per 1000 admissions in 2007. Additionally, there has been a constant rise in PAC use to 12 PAC per 1000 admissions in 2014 (*p* < 0.001). Interestingly, there has been a gradual decline in the use of PAC in patients with CS, from 123 PAC used per 1000 admissions in 2004 to 78 PAC per 1000 admissions in 2014 (*p* < 0.001). Moreover, patients with PAC had increased hospital costs, length of stay, and mechanical circulatory support use. In patients with HF, PAC use was associated with a higher mortality. In those with CS, PAC was associated with a lower mortality and lower in-hospital cardiac arrest; the paradox was also present after propensity score-matching.

Nevertheless, it should be addressed that PAC-guided therapy improved filling pressures and reduced the rates of atrium/brain natriuretic peptides (ANP/BNP) and patients’ quality of life expressed by trade-off protocol. Moreover, its use did not have any impact on overall mortality and, last but not least, its implementation was not assessed in a triage of patients with cardiogenic shock or eligible for ventricular assist device (VAD)/orthotopic heart transplantation (OHT). On the other hand, Tehrani et al. proposed a different, team-based therapy for CS on the base of RHC data. This approach enabled a significant reduction in mortality from 47% in 2016 to 76.6% in 2018 (*p* < 0.01) [89]. Ma et al. re-evaluated publicly accessible data on the previously mentioned ESCAPE trial to discriminate the potential prognostic role of the invasively derived parameters [90]. Their post-hoc analysis revealed that a group of patients characterized by a single hemodynamic target, defined as PCWP + RAP ≥ 30 mmHg (congestion index), was associated with poor prognosis and increased relative risk (RR) of death (RR 5.76), death-or-transplantation (DT) (RR 4.92), and death-or-rehospitalization (DR) (RR 1.80) at six months follow-up. Moreover, this group had 45.3% mortality, 54.7% DT, and 84.9% DR at six months, contrasting with the cohort of PCWP + RAP ˂ 30 mmHg, which had 8.7% mortality, 13.0% DT, and 58.7% DR at six months. These observations suggest that the PAC-guided treatment of filling pressures and volume overload in the specific groups of most demanding patients with end-stage heart failure or after VADs implantations needs to be assessed in randomized controlled trials [91,92,93,94,95], especially given that the progress in the management of HF through the last decade, including pharmacology and technical possibilities, has been promising [96]. To see the recapitulative table with the major studies and findings, please refer to Table 2, depicted below.

### 3.3. Position of the RHC in the Diagnostic Algorithm of Pulmonary Hypertension

RHC is currently considered a gold standard in the assessment of PH, which by definition is associated with the elevation of PAPm above 25 mmHg at rest [1,2,97]. Clinical investigation, electrocardiogram, chest radiograph, and echocardiography are the first-line examinations in this entity; however, RHC is obligatory not only to establish the definite diagnosis but also to differentiate between the subtypes of PH and prognosticate response to treatment and survival rate. Guidelines, along with the World Health Organization, classify PH into five groups of etiologic entities characterized by different hemodynamic profiles.

**Figure 3:**
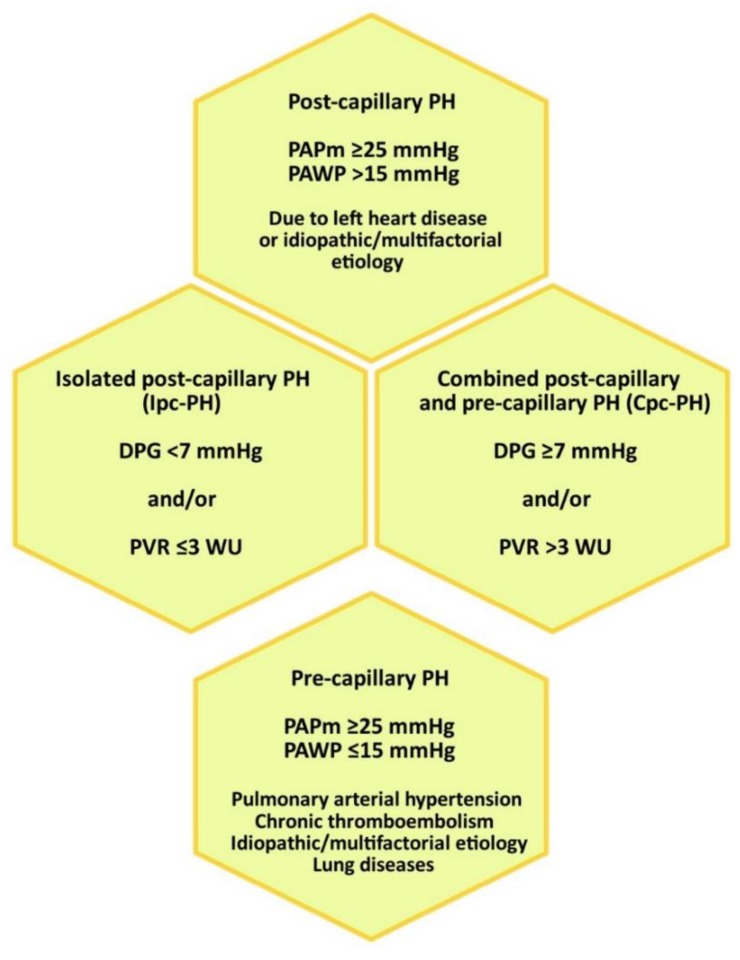
The clinical characteristics of pulmonary hypertension on the base of current guidelines. PH—pulmonary hypertension, PAPm—pulmonary artery pressure mean, PAWP—pulmonary artery wedge pressure, DPG—diastolic pressure gradient, PVR—pulmonary vascular resistance.

Pre-capillary PH is characterized by PAPm ≥ 25 mmHg, and PAWP ≤ 15 mmHg may occur in clinical groups 1, 3–5, where groups 2 and 5 in special conditions consist of different forms of post-capillary PH, which is characterized by PAPm ≥ 25 mmHg and PAWP > 15 mmHg. It can be further divided into isolated postcapillary PH with DPG (diastolic pressure gradient) < 7 mmHg and/or PVR ≤ 3 Wood Units (WU), or a combined form of either pre–and post-capillary component with DPG ≥ 7 mmHg and/or PVR > 3 WU. It needs to be underlined that the role of DPG in the diagnosis of PH has decreased recently, since it is challenging to prove its influence on a patient’s prognosis [98,99]. The physiological characteristics of pulmonary hypertension presented from the clinical point of view are depicted in Figure 3.

The performance of vasorecactivity testing is recommended in the arterial form of PH, where it contributes significantly to the choice of the treatment regimen [100]. The encouraged vasoreactive agent is nitric oxide (NO), with a dose of 10–20 ppm, and the test is interpreted as reactive, whether the reduction in mean PAP is 10 mmHg or higher. However, the absolute value should be 40 mmHg or less [1,99]. Patients presenting significant reductions in pulmonary pressures in vasorreactivity testing during RHC may benefit from calcium channel blockers therapy, although the absolute number of these cases is quite restricted [101,102].

Some experienced heart centers use the vasoreactivity testing in heart failure scenario associated with type 2 PH as a part of the integral stratification prior to OHT. Paradoxically, increased PVR above 2.5 WU was associated with unfavorable prognosis only if it was associated with a decrease in systolic blood pressure below 85 mmHg during vasoreactive testing with sodium nitroprusside [103,104]. These data, although not standardized, sustain the theoretical assumptions that reversibility testing has the prognostic competence to discriminate the patients prone to developing acute right ventricle failure after OHT.

## 4. Conclusions

RHC, although present in experimental physiology and medicine since the beginning of the seventeenth century, still has safety concerns. These, however, are predominantly related to its depersonalized, randomly assigned utilization, regardless of its risk-to-benefit ratio to the patients. RHC is indispensable in PH diagnosis settlement, treatment monitoring, and decision-making. Moreover, it is imperative prior to OHT to stratify the risk of early post-operative RV failure, which might be useful in the end-stage heart failure group of patients (including VADs) in the optimization of pharmacotherapy.

## Figures and Tables

**Table 1:** Basic definitions and the range of normal values.

Parameter	Abbreviation	Normal Value	Definitions/Comments
Heart rate	HR	60–100 bpm	number of beats per minute
Stroke volume	SV	60–100 mL/beat	the amount of blood pumped by each ventricle of the heart during contraction
Cardiac output	CO	4.0–8.0 L/min	volume of blood being pumped within the minute
Body surface area	BSA	1.6–1.9 m^2^	the measured or calculated surface area of a human body
Cardiac index	CI	2.5–4.0 L/min/m^2^	CO/BSA
Central Venous Pressure	CVP	2–6 mmHg	superior vena cava pressure—might be used to estimate preload and right atrial pressure
Right atrial pressure	RAP	2–6 mmHg	usually RAP~CVP
Pulmonary artery pressure (systolic/diastolic/mean) (s/d/m)	PAPs/d/m	15–30/8–15/9–18 mmHg	assessed by a Swan-Ganz catheter placed in either RPA or LPA
Pulmonary artery wedge pressure	PAWP	6–12 mmHg	the inflated balloon of the catheter tip passively transmits the pressure of LA
Transpulmonary gradient	TPG	≤12 mm Hg	PAPm—PAWPm
Pulmonary vascular resistance	PVR	<3.125 WU	PAPm—PAWPm/CO
Diastolic pulmonary gradient	DPG	<7 mmHg	PAPd—PAWPm
Systemic vascular resistance	SVR	10–15 WU	SBPm—RAPm/CO

Table legend: SBPm–systemic blood pressure mean, WU–Wood Units.

**Table 2:** Summary of the most important studies describing the role pulmonary artery catheters.

Author, Reference Number, and the Year of Publication	Number of Patients and the Time of Observation	Primary Condition Diagnosed/Treated	Results and Main Findings
Connors [76], 1996 “SUPPORT”	5735 pts, 180 days	Severe illness. RHC group versus no RHC group: Acute respiratory failure: 589 (27%) versus 1200 (34%) Multiorgan system failure: 1235 (57%) versus 1245 (35%) Congestive heart failure: 209 (10%) versus 247 (7%) Other: 151 (7%) versus 859 (24%)	1. Patients with RHC had an increased 30-day mortality (odds ratio, 1.24; 95% confidence interval, 1.03–1.49); 2. The mean cost (25th, 50th, 75th percentiles) per hospital stay was $49 300 ($17,000, $30,500, $56,600) with RHC and $35,700 ($11,300, $20,600, $39,200) without RHC; 3. Mean length of stay in the ICU was 14.8 (5, 9, 17) days with RHC and 13.0 (4, 7, 14) days without RHC; 4. Conclusions: After adjustment for treatment selection bias, RHC was associated with increased mortality and increased utilization of resources.
Wheeler [77], 2006	1000 pts, 60 days	Acute lung injury (ALI)	5. Patients randomized into PAC versus CVC guided therapy; 6. The rates of death during the first 60 days before discharge home were similar in the PAC and CVC groups (27.4% and 26.3%, respectively; *p* = 0.69); 7. Complications were uncommon and were reported at similar rates in each group: 0.08 ± 0.01 per catheter inserted in the PAC group and 0.06 ± 0.01 per catheter inserted in the CVC group (*p* = 0.35); 8. Conclusions: The routine use of PAC in the ALI group of pts is discouraged.
Harvey [78], 2005 “PAC-Man”	1014 pts, 90 days	Acute respiratory failure (13%), Multiorgan dysfunction (65–66%), Decompensated heart failure (11%), Other (10–11%)	9. No difference in hospital mortality between patients managed with or without a PAC (68% (346 of 506) versus 66% (333 of 507), *p* = 0.39); 10. No clear evidence of benefit or harm by managing critically ill patients with a PAC; 11. Nearly 10% of pts in the PAC arm suffered from the complications (none of these were fatal).
Ramsey [82], 2004	13,907 pts, 7–8 days	Aortocoronary bypass for heart revascularization—any type	12. The relative risk of in-hospital mortality was 2.10 for the PAC group compared with the patients who did not receive a PAC (95% confidence interval (CI), 1.40 to 3.14; *p* < 0.001); 13. The mortality risk was significantly higher in hospitals with the lowest third of PAC use (odds ratio, 3.35; 95% Cl, 1.74 to 6.47; *p* < 0.001); 14. Not significantly increased in the highest two thirds of users (odds ratio, 1.62; 95% Cl, 0.99 to 2.66; *p* = 0.09); 15. Days spent in critical care were similar, although total length of hospital stay was 0.26 days longer in the PAC group (*p* < 0.001); 16. Hospital costs were $1402 higher in the PAC group.
Binanay [85], 2005 “ESCAPE”	433 pts, 180 days	Severe symptomatic heart failure despite recommended therapies	17. The use of the PAC did not significantly affect the primary end point of days alive and out of the hospital during the first six months (133 days versus 135 days; hazard ratio (HR), 1.00 (95% confidence interval (CI), 0.82–1.21); *p* = 0.99); 18. Mortality (43 patients (10%) versus 38 patients (9%); odds ratio (OR), 1.26 (95% CI, 0.78–2.03); *p* = 0.35), or the number of days hospitalized (8.7 versus 8.3; HR, 1.04 (95% CI, 0.86–1.27); *p* = 0.67); 19. In-hospital adverse events were more common among patients in the PAC group (47 (21.9%) versus 25 (11.5%); *p* = 0.04); 20. There were no deaths related to PAC use; 21. No difference for in-hospital plus 30-day mortality (10 (4.7%) versus 11 (5.0%); OR, 0.97 (95% CI, 0.38–2.22); *p* = 0.97);
Sionis [86], 2019 “CardSchock”	219 pts, 82 (37.4%) received PAC, 30 days	Patients with cardiogenic shock included in the CardShock Study	22. Cardiogenic shock patients who managed with a PAC received more frequent treatment with inotropes and vasopressors, mechanical ventilation, renal replacement therapy, and mechanical assist devices (*p* < 0.01); 23. Overall 30-day mortality was 36.5%. Pulmonary artery catheter use did not affect mortality even after propensity score matching analysis (hazard ratio = 1.17 (0.59–2.32), *p* = 0.66).
Doshi [87], 2018	6,645,363 pts, in hospital stay between 3–17 days	HFrEF—3,225,529 hospitalizations HFpEF—3,419,834 hospitalizations	24. Per 1000 hospitalizations, the use of PAC declined from 2005 to 2010 in both HFrEF (12.9 to 7.9, *p* < 0.001) and HFpEF (12.9 to 5.5, *p* < 0.001); 25. From 2010 to 2014, the use of PAC per 1000 hospitalizations increased in both HFrEF (7.9 to 9.7, *p* < 0.001) and HFpEF (5.5 to 6.7, *p* < 0.001); 26. The temporal decline in risk-adjusted mortality during the study period for HFrEF (odds ratio, 3.93 in 2005–2006 to 2.7 in 2013–2014, *p* < 0.001) and HFpEF (odd ratio, 2.72 in 2005–2006 to 2.62 in 2013–2014, *p* < 0.001); 27. The length of stay and cost were significantly higher with PAC use in both HFrEF and HFpEF.
Hernandez [88], 2019	9,431,944 pts, in hospital stay between 2–20 days	HF (*n* = 8,516,528) index manifestation CS (*n* = 915,416) developed during the index hospitalization	28. Overall, patients with PAC had increased hospital costs, length of stay, and mechanical circulatory support use; 29. In patients with HF, PAC use was associated with higher mortality (9.9% versus 3.3% OR 3.96 *p* < 0.001); 30. In those with CS, PAC was associated with lower mortality (35.1% versus 39.2% OR 0.91 *p* < 0.001); 31. And lower in-hospital cardiac arrest (14.9% versus 18.3% OR 0.77 *p* < 0.001); 32. This paradox persisted after propensity score matching.
Tehrani [89], 2019	204 consecutive pts, 30-day survival	CS	33. Compared with a 30-day survival of 47% in 2016, the 30-day survival in 2017 and 2018 increased to 57.9% and 76.6%, respectively (*p* < 0.01); 34. Independent predictors of 30-day mortality were age ≥ 71 years, diabetes mellitus, dialysis, ≥36 h of vasopressor use at time of diagnosis, lactate levels ≥ 3.0 mg/dL, CPO < 0.6 W, and PAPi < 1.0 at 24 h after diagnosis and the implementation of therapies; 35. Either 1 or 2 points were assigned to each variable, and a three-category risk score was determined: 0 to 1 (low), 2 to 4 (moderate), and ≥5 (high); 36. Conclusions: A standardized team-based approach may improve CS outcomes; 37. A score incorporating demographic, laboratory, and hemodynamic data may be used to quantify risk and guide clinical decision-making for all phenotypes of CS.

Table legend: PAC—pulmonary artery catheter, CVC—central venous catheter, ALI—Acute Lung Injury, HR—Hazard ratio, OR—Odds ratio, CI—confidence interval, HFrEF/HFpEF—heart failure with reduced/preserved ejection fraction, CS—cardiogenic shock, CPO—cardiac power output, PAPi—pulmonary arterial pulsatility index.
